# Does the Bilingual Advantage in Cognitive Control Exist and If So, What Are Its Modulating Factors? A Systematic Review

**DOI:** 10.3390/bs9030027

**Published:** 2019-03-13

**Authors:** Maurits van den Noort, Esli Struys, Peggy Bosch, Lars Jaswetz, Benoît Perriard, Sujung Yeo, Pia Barisch, Katrien Vermeire, Sook-Hyun Lee, Sabina Lim

**Affiliations:** 1 Research Group of Pain and Neuroscience, Kyung Hee University, Seoul 130-701, Korea; sh00god@khu.ac.kr; 2 Brussels Institute for Applied Linguistics, Vrije Universiteit Brussel, 1050 Brussels, Belgium; Esli.Struys@vub.be; 3 Psychiatric Research Group, LVR-Klinik Bedburg-Hau, 47511 Bedburg-Hau, Germany; p.bosch@donders.ru.nl; 4 Donders Institute for Brain, Cognition and Behaviour, Radboud University, 6525 Nijmegen, The Netherlands; 5 Behavioural Science Institute, Radboud University, 6525 Nijmegen, The Netherlands; L.Jaswetz@psych.ru.nl; 6 Department of Medicine, Neurology, University of Fribourg, 1700 Fribourg, Switzerland; benoit.perriard@unifr.ch; 7 College of Oriental Medicine, Sang Ji University, Wonju 26339, Korea; pinkteeth@hanmail.net; 8 Institute of Experimental Psychology, Heinrich Heine University, 40225 Düsseldorf, Germany; Pia.Barisch@uni-duesseldorf.de; 9 Department of Communication Sciences and Disorders, Long Island University (LIU) Brooklyn, Brooklyn, NY 11201, USA; katrien.vermeire@liu.edu

**Keywords:** bilingual advantage, bilingualism, cognitive control, individual differences, longitudinal studies, methodology

## Abstract

Recently, doubts were raised about the existence of the bilingual advantage in cognitive control. The aim of the present review was to investigate the bilingual advantage and its modulating factors. We searched the Medline, ScienceDirect, Scopus, and ERIC databases for all original data and reviewed studies on bilingualism and cognitive control, with a cut-off date of 31 October 2018, thereby following the guidelines of the preferred reporting items for systematic reviews and meta-analysis (PRISMA) protocol. The results of the 46 original studies show that indeed, the majority, 54.3%, reported beneficial effects of bilingualism on cognitive control tasks; however, 28.3% found mixed results and 17.4% found evidence against its existence. Methodological differences seem to explain these mixed results: Particularly, the varying selection of the bilingual participants, the use of nonstandardized tests, and the fact that individual differences were often neglected and that longitudinal designs were rare. Therefore, a serious risk for bias exists in both directions (i.e., in favor of and against the bilingual advantage). To conclude, we found some evidence for a bilingual advantage in cognitive control; however, if significant progress is to be made, better study designs, bigger data, and more longitudinal studies are needed.

## 1. Introduction

The majority of individuals in the world speak at least two languages [1]. In several countries, like the Netherlands, Belgium, etc., at least three foreign languages are taught to children in school. Moreover, due to migration patterns, many cities have become highly multilingual, and individuals encounter several foreign languages at work or in their leisure time. In a more global world, due to the development of the internet and as a result of an increase in international travel for work or tourism, the knowledge of foreign languages is further increasing [2].

Of course, the more one uses a second language (L2) and comes into contact with that language, the better those language skills will be; i.e., people start to improve their L2 reading, speaking, writing, and listening skills. Age seems to be an important factor in L2 learning. In general, children learn foreign languages faster, retain them better, and most often speak them with near-native pronunciation [3], although several morphosyntactic categories are mastered faster by adolescents and adults than by young learners. Whether a “critical period” in L2 learning exists [4], what the exact nature (the strong version [4] or the weak version [5]) of that critical period is, and with which cut-off age this goes away, i.e., 17 years [6], 7 years [4], or 3 years [7], has been the subject of a long and vivid ongoing debate [8,9,10]. Despite the possible existence of a critical period in L2 learning, individuals are also able to learn foreign language skills later in life [11]. Moreover, regardless of the onset age of L2 learning, individual differences seem to exist in the success of that learning [11]. Individual differences in such factors as aptitude, motivation, learning strategies, learning styles, meta-linguistic awareness, personality traits (e.g., extraversion), etc. have been suggested to play roles in L2 learning [11].

Interestingly, however, bilingualism was found to have beneficial effects not only in the expected linguistic domains, but also in other domains, such as attention [12], working memory [13], and cognitive control [14]. In the literature, this effect is generally referred to as the “bilingual advantage” [15]. With the term bilingual advantage, what is meant is the skill areas in which bilinguals outperform monolinguals. In the present review, the specific focus will be the process of cognitive control in bilinguals. Cognitive control is defined as “the coordination and regulation of thoughts to respond appropriately to salient stimuli in the environment and to maintain focus on goal-directed behavior” [16]. It includes inhibitory control, attention, working memory, cognitive flexibility, planning, reasoning, and problem solving [16]. Note that in daily practice, the bilingual speaker has to process and manage two (or more) language systems [17]. In order to perform this task successfully, the bilingual speaker has to suppress interference from the nontarget language(s) while speaking or recognizing the target language [18]. In addition, the bilingual speaker needs to be able to produce or recognize language switches when changing from one language to the other [19]. This extra training in cognitive control skills in bilinguals compared to monolinguals is thought to be the reason bilinguals have this (bilingual) advantage in cognitive control [20].

However, the questions remain as to whether this bilingual advantage is the same for all bilinguals and why some studies fail to find it [21,22,23]. Thus, another rising question is which factors modulate the bilingual advantage in cognitive control. Two types of factors, individual and methodological, may explain the varying findings of the studies conducted so far. Regarding individual modulating factors, earlier studies showed that ethnic, as well as socioeconomic, background did modulate the bilingual advantage [24]. Regarding methodological factors, we must stress that the studies conducted until now used various kinds of tasks, as well as different groups of participants (different ages, different kinds of bilinguals). However, the ways in which those methodological variations impact the bilingual advantage in cognitive control are not clear. Moreover, this is also true for the individual factors; so far, the exact effects of these individual factors on the bilingual advantage remain undetermined.

Therefore, the major aim of the present study was to provide an overview of studies published so far on bilingualism and cognitive control, as well as their findings, in an effort to determine whether or not a bilingual advantage in cognitive control really exists. Furthermore, the focus was on individual, as well as methodological, factors such as socioeconomic status [24], cognitive capacity [25], culture [24], age, task used, etc. that might modulate the bilingual advantage in cognitive control. The expectation was that bilinguals perform better than monolinguals on cognitive control tasks. Thus, we expected the majority of studies to find a bilingual advantage in cognitive control. Moreover, we hypothesized that individual, as well as methodological, factors affect the bilingual advantage in cognitive control.

## 2. Materials and Methods

### 2.1. Search Strategies

A systematic review on bilingualism and cognitive control was conducted with a particular interest in the factors affecting this beneficial bilingualism effect. In this study, with a cut-off date of 31 October 2018, the Medline (https://www.ncbi.nlm.nih.gov/pubmed/), ScienceDirect (https://www.sciencedirect.com/), Scopus (https://www.elsevier.com/solutions/scopus), and ERIC (https://eric.ed.gov/) databases were searched for all original data and review studies on bilingualism and cognitive control. Thereby the guidelines of the preferred reporting items for systematic reviews and meta-analysis (PRISMA) protocol [26] were followed. The following combinations of keywords were used: “bilingual advantage” AND “cognitive control”; “bilingualism” AND “Simon task”; “bilingualism” AND “ANT task”; “bilingual advantage” AND “flanker task”; “bilingualism” AND “cognitive control”; “bilingual advantage”; and “multilingualism” AND “cognitive control”.

### 2.2. Study Selection and Data Extraction

First, three investigators (P.B., B.P., and L.J.) independently searched the Medline, ScienceDirect, Scopus, and ERIC databases. Then, three different researchers (M.N., E.S., and S.Y.) independently selected the relevant studies and extracted the data. The selection of relevant studies was conducted based on previously determined inclusion and exclusion criteria. To be considered for inclusion, the study had to be published in a peer-review format. Furthermore, the cognitive control performance of bilinguals compared to monolinguals had to have been investigated in the study. In addition, only studies involving healthy participants, data papers, and review papers were selected, while case studies, commentaries, and other formats were excluded. Finally, another inclusion criterion was that both monolingual and bilingual data should be presented in the selected study.

In some cases, the original authors were contacted in order to gain more information and to decide whether the study was relevant or not. The following data were used in the present review: The authors and the title of study; the journal in which the study had been published and the publication year; the numbers of bilingual and monolingual subjects that participated in the study; information regarding the experimental tasks and methodology that had been used; the risk of bias (this was assessed indirectly, based on previous review studies); the results of the study, especially whether a bilingual advantage was found or not; and finally, the conclusions that had been drawn by the authors of the study. Moreover, in cases of disagreement, four different researchers (P.B.A., S.L., K.V., and S.H.L.) were asked to evaluate the study in question for inclusion in this review. Finally, in all cases, consensus was eventually reached among all nine authors.

## 3. Results

### 3.1. General Results

**Figure 1:**
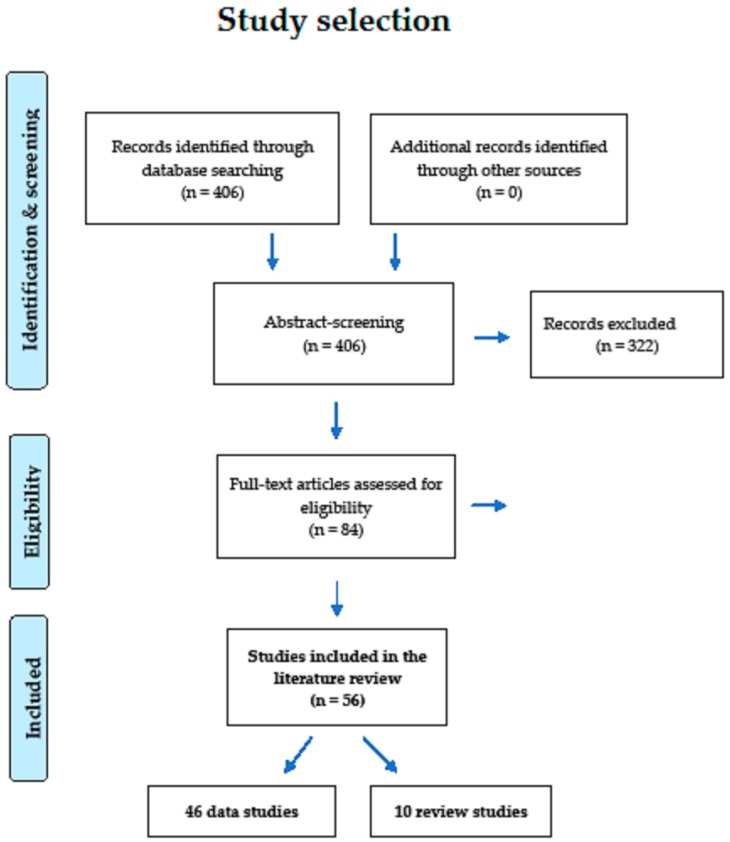
Overview of the selection process for the studies included in this review.

**Figure 2:**
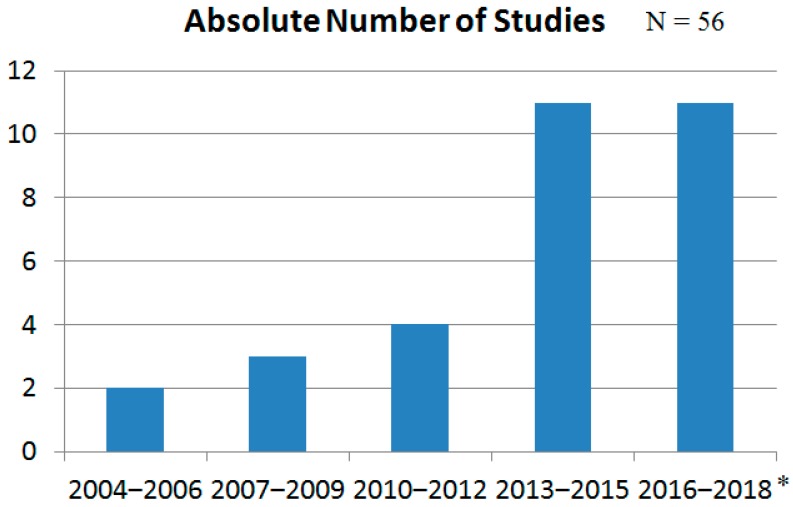
Overview of the growth in the number of bilingual (original and review) studies on cognitive control over the period from 1 January 2004 to 31 October 2018. Over the past six years, a clear increase in the number of bilingual studies on cognitive control can be seen. * = Only studies that were published on or before 31 October 2018 were included.

As can be seen in Figure 1, our search found 406 articles, of which 84 were relevant. Fifty-six of those 84 satisfied the inclusion criteria and were eligible for inclusion in this review. Of the 56, 46 were original studies [7,14,21,27,28,29,30,31,32,33,34,35,36,37,38,39,40,41,42,43,44,45,46,47,48,49,50,51,52,53,54,55,56,57,58,59,60,61,62,63,64,65,66,67,68,69] and 10 were review/meta-analysis studies [70,71,72,73,74,75,76,77,78,79]. The bilingual studies were conducted on several continents, with 23 (41.1%) having been conducted in North America (particularly in Canada) [14,21,27,29,30,31,32,34,38,45,48,53,54,55,60,63,69,70,71,72,74,75,78], 5 (8.9%) having been conducted by a North American/European collaboration [36,42,47,50,58], 2 (3.6%) having been conducted by a North American/Asian collaboration [54,61], 1 (1.8%) having been conducted by a North American/European/Asian collaboration [28], 18 (32.1%) having been European studies [33,35,37,39,41,43,46,49,51,52,57,64,66,67,68,73,76,77], 1 (1.8%) having been conducted by a European/Australian collaboration [40], 2 (3.6%) having been conducted by a European/Asian collaboration [7,79], and 4 (7.1%) having been Asian studies [44,59,61,65]. To date, African or Latin American studies on bilingualism and cognitive control have still not been published. When all original studies included in this review are taken together, 2692 bilingual participants were involved, of whom 601 were children and 2091 were adults. Moreover, clearly, more studies are conducted on bilingual adults (n = 39; especially on young adults) than on bilingual children (n = 7). In the past six years, a clear increase in the number of bilingual studies on cognitive control can be seen. Figure 2 shows the absolute numbers of studies over the period from 1 January 2004, until 31 October 2018, in intervals of three years.

**Figure 3:**
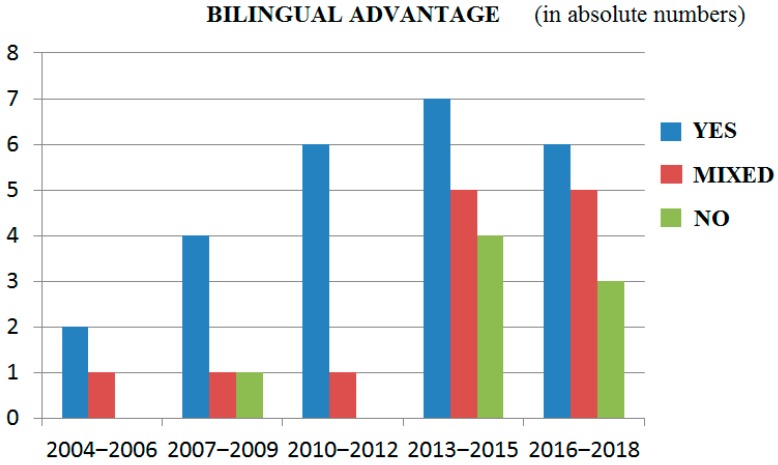
Overview of the absolute numbers of studies that found evidence in favor of a bilingual advantage in cognitive control, that found mixed results, and that found evidence against the existence of a bilingual advantage in cognitive control during the last 15 years. The results are specified for five three-year periods over the last 15 years.

As can be seen in Table 1, the general results of the present review show that the majority, 54.3% (25/46), of the original studies indeed found a bilingual advantage in cognitive control, 28.3% (13/46) found mixed results, and 17.4% (8/46) found evidence against the existence of a bilingual advantage. When the age of the included participants was taken into account, more evidence in favor of the existence of a bilingual advantage in cognitive control was found in adults. For the adult bilinguals, 56.4% (22/39) of the original studies indeed found a bilingual advantage in cognitive control, whereas 28.2% (11/39) found mixed results and 15.4% (6/39) found evidence against the existence of that advantage. Compared to that, in studies investigating children, 42.8% (3/7) of the original studies found results in favor of the existence of a bilingual advantage, 28.6% (2/7) found mixed results, and 28.6% (2/7) found evidence against its existence. In general, as can be seen in Figure 3, the evidence in favor of the existence of a bilingual advantage was stronger in the earlier studies conducted in the period from 2004 to 2012, whereas more studies showing mixed findings and evidence against the existence of a bilingual advantage were found in more recent years (from 2013 until October 2018).

Different tasks have been used to test the bilingual advantage in cognitive control; among them, the Simon task [80], the attention network test [81], Flanker tasks [82], the Stroop task [83], and switching tasks [36] have been most frequently used to test the bilingual advantage in cognitive control. Of the 46 original studies implemented in the present review, 23 used the Simon task, 5 the attention network test, 9 Flanker tasks, 9 the Stroop task, and 7 a switching task; moreover, in 20 original studies, other experimental tasks were used: e.g., verbal fluency [84], interpretation, a judgment task [53], an N-back task [85], a reading task, a picture–word identification task [63], the Wisconsin card sorting test [86], the Tower of London task [87], the digit span task [88], the Hebb repetition paradigm [89], Luria’s tapping task [90], the opposite worlds task [91], the reverse categorization task [92], the sustained attention to response task [93], the trail making test [94], and the dichotic listening task [95]. Please note that some studies used more than one experimental task, and as a result, the total number of experimental tasks is higher than the total number of original studies.

### 3.2. Bilingual Advantage in Children

Age is known to be an important factor in learning an L2, as well as acquiring cognitive control skills. Therefore, the cognitive control results that were collected from original studies on children will be presented first, after which the results for bilingual adults will be presented. As Table 1 shows, Engel de Abreu and colleagues [42] used various cognitive tasks to test 40 children and found, in comparison to monolingual children, bilingual children had a bilingual advantage in cognitive control but not in the other domains. This was also true when controlling for socioeconomic status and cultural factors. Note that Engel de Abreu and colleagues [42] tested children from a low socioeconomic status. Bialystok and colleagues [36] also found evidence in favor of a bilingual advantage in cognitive control. The 56 bilingual children performed better than the monolingual children on three out of the four executive functioning tasks. In addition, Poarch and Bialystok [54] found in their study that the bilingual children outperformed monolingual children on the conflict trials in the flanker task [82].

By contrast, Morton and Harper [30] tested monolingual and bilingual children on the Simon task [80] and found no evidence of an advantage for bilingual children compared to monolingual children when socioeconomic status and ethnicity were taken into account. The monolingual children and the bilingual children performed the same. The only difference that was found in that study was that children from families with higher socioeconomic status were advantaged relative to children from families with lower socioeconomic status. Duñabeitia and colleagues [46] also failed to find evidence for the existence of a bilingual advantage. They used a verbal and a nonverbal Stroop task [83] to test 252 bilingual and 252 monolingual children and found similar performances for both groups on simple inhibitory tasks. Struys and colleagues [7] conducted research on two different bilingual groups: A group of simultaneous bilingual children (meaning children who had become bilingual by learning two languages from birth) and a group of early bilingual children (meaning children who had learned their L2 from age three onward). In line with the bilingual advantage hypothesis, they found a higher global accuracy score for the simultaneous bilingual children; however, surprisingly, they did not find faster mean reaction times for those children compared to the early bilingual children. In another study, Struys and colleagues [66] tested two groups of bilingual children, one of younger children and the other of older children, and two groups of monolingual children, one of younger children and the other of older children, on the Simon task [80] and the Flanker task [82]. The results showed no differences between the bilinguals and the monolinguals. Interestingly, however, only the bilinguals were found to show a significant speed–accuracy trade-off across tasks and age groups.

### 3.3. Bilingual Advantage in Adults

#### 3.3.1. Behavioral Results

As Table 1 shows, the majority of studies reported a bilingual advantage in cognitive control for adult bilinguals. In those studies, bilingual adults were compared with monolingual adults in their performances on cognitive control tasks. Bialystok and colleagues [27], for instance, found that controlled processing was carried out more effectively by bilingual adults than by monolingual adults and that bilingualism seemed to help to offset age-related losses in certain executive processes. In another study, Bialystok [29] found that bilingual adults were faster than monolingual adults in conditions that required the most controlled attention to resolve conflict. In order to investigate whether age had affected the bilingual advantage results, Bialystok and colleagues [31] conducted a study in which both young and older monolingual and bilingual adults were included. They found that bilingual adults performed better than monolingual adults on the executive functioning tasks and that this advantage was stronger in the older group. Bialystok and DePape [34] found that bilingual adults outperformed monolingual adults in executive control in another study, and in line with previous findings. This was also what Schroeder and colleagues [60] found; bilingual adults and bilingual musicians outperformed monolingual adults and monolingual musicians. In addition, monolingual musicians showed improved executive control scores compared to monolingual adults. Garbin and colleagues [37] found a reduced switching cost in bilingual adults. Costa and colleagues [33] found that bilingual adults had more efficient attentional mechanisms than monolingual adults. Moreover, in another study, Costa and colleagues [35] found that bilingual adults were faster than monolingual adults under high-monitoring conditions, supporting the hypothesis that bilingualism may affect the monitoring processes involved in executive control. Luo and colleagues [38] also found that bilingual adults showed enhanced executive control, but they found this result on a verbal fluency task. In line with previous findings, Teubner-Rhodes and colleagues [58] found that bilingual adults performed better than monolingual adults on a high-conflict task. This is also what Desideri and Bonifacci, [64] found; bilingual adults showed a better conflict performance than monolingual adults and overall faster reaction times. Cox and colleagues [57] also found evidence supporting the ‘bilingual advantage in cognitive control’ hypothesis. L2 learning was found to be related to better conflict processing; moreover, neither initial childhood ability nor social class was found to be a modulating factor. Furthermore, Marzecová and colleagues [43] found that bilingualism positively influenced mechanisms of cognitive flexibility. Blumenfeld and Marian [14] found evidence for a bilingual advantage in cognitive control where bilingualism may be especially likely to modulate cognitive control mechanisms resolving the stimulus–stimulus competition between two dimensions of the same stimulus. Macnamara and Conway [45] made an interesting new contribution to the research field when they conducted a study with a longitudinal design, in which they tested bilingual participants twice. They found that the bilingual adults had improved cognitive abilities associated with managing bilingual demands after two years, tapping more directly into the ongoing process of the bilingual advantages in cognitive control.

However, not all bilingual adults have the same bilingual background; i.e., one can acquire the L2 from birth onwards; one can become highly proficient in the L2 or less proficient in later life; and so on. Thus, the question is, do all bilinguals show a bilingual advantage or is this only the case for some specific subgroup or subgroups of bilinguals? In order to investigate whether differences in the bilingual advantage exist within a group of bilinguals, researchers must investigate specific subgroups of bilinguals. Emmorey and colleagues [32], for instance, made a specification in the kind of bilingual participants and tested unimodal (individuals fluent in two spoken languages) versus bimodal (individuals who are fluent in a signed and a spoken language) bilinguals. They found a bilingual advantage for the unimodal bilinguals but not for the bimodal bilinguals when compared to monolinguals. Unimodal bilinguals were found to have faster response times than monolinguals. Tao and colleagues [40] specifically looked at the age of acquisition of their bilingual participants and found that both early and late bilinguals had an advantage in conflict resolution compared to monolinguals. The greatest advantage, however, was found for early bilinguals. Woumans and colleagues [52] also made a specification in the kind of bilingual participants: They tested three different bilingual groups, unbalanced bilinguals (individuals who speak two languages but are more skilled in one language than in the other), balanced bilinguals (individuals who have equal proficiency in both the native language and the L2), and interpreters; a monolingual group was also included in the study. Evidence in favor of the bilingual advantage in cognitive control was found in all three bilingual groups. Dong and Liu [59] reported that bilinguals with interpreting experience showed improvements in switching and updating performance, while bilinguals with translating experience showed only marginally significant improvements in updating. Thus, processing demand was found to be an important factor modulating the bilingual advantage.

Hsu [44] made a clear distinction between early balanced bilingual and trilingual individuals. Monolingual, bilingual, and trilingual participants were tested in that study. Hsu [44] found that for the trilingual participants, a clear advantage in inhibitory and attentional control existed while for the bilingual participants, only an advantage in inhibitory control was found when compared to the monolinguals. In a recent study by Hsu [61], balanced and unbalanced bilinguals were found to be better than monolinguals on the noncontextual single-character reading task (regardless of their first language background), but not on the contextual multiword task. Moreover, Hsu [61] found that unbalanced bilinguals performed better on the noncontextual task than both the balanced bilingual and monolingual groups. In other words, these results explain how the effects of bilingualism and cross-linguistic similarity dynamically interplayed depending on the task contexts and the relative degrees of using the mother tongue and L2 [61]. Xie [65] looked more closely at the level of L2 proficiency. The degree of L2 proficiency affected conflict monitoring but not inhibition or mental set shifting.

However, not all studies found evidence in favor of a bilingual advantage in adults. Van der Linden and colleagues [68], for instance, found no support for the existence of a bilingual advantage for interpreters and L2 teachers who were highly proficient in their L2. Kirk and colleagues [49] also found no evidence for a bilingual or bidialectal advantage in executive control in their study on older adults. Coderre and van Heuven [47] found mixed results because they only found global response time effects in their data. On the other hand, Goral and colleagues [55] found that the results for the dominant bilinguals supported the bilingual advantage hypothesis, whereas the results for balanced bilinguals showed age-related inhibition decline, which goes against the hypothesis. Yudes and colleagues [41] found mixed results, as well. The interpreters that were highly skilled bilinguals outperformed unbalanced, late bilinguals and monolinguals in cognitive flexibility but not in inhibition. This finding of overall faster response times in bilinguals was also found in a study by Naeem and colleagues [67]; however, that result disappeared when they controlled for socioeconomic status. The results collected by Paap and Greenberg [21] showed no evidence for consistent cross-task advantages in executive processing for bilinguals compared to monolinguals; this was also found in a study by Kousaie and colleagues [48]. Sometimes, bilingual advantages are visible in the data for one specific task, but they are not seen in the data for another task measuring the same executive processing skills.

#### 3.3.2. Neuroimaging Results

Hervais-Adelman and colleagues [51] studied the effect of L2 proficiency. They conducted a study on highly proficient multilinguals. In their functional magnetic resonance imaging (fMRI) study, a clear dissociation of specific dorsal striatum structures in multilingual language control was found. These areas are known to be involved in nonlinguistic executive control, supporting the bilingual advantage hypothesis. Blanco-Elorrieta and Pylkkänen found mixed results in their magnetoencephalography (MEG) study [56] on highly proficient bilinguals; their neuroimaging results indeed showed evidence for the hypothesis that language control is a subdomain of general executive control in production, as the bilingual advantage hypothesis would suggest. In a second MEG study [62], Blanco-Elorrieta and Pylkkänen showed that the bilingual advantage effects are only visible in switching tasks when bilinguals need to control their languages according to external cues and not when they can voluntarily switch. Ansaldo and colleagues [50] also found mixed results in their fMRI study. On the one hand, the neuroimaging results supported the bilingual advantage hypothesis, but on the other hand, the behavioral results showed no support for any bilingual advantages in cognitive control. Kousaie and Phillips [63] also found mixed results in their electroencephalography (EEG) study. Group differences in electrophysiological results on all three cognitive control tasks between bilinguals and monolinguals were found, which is what the bilingual advantage hypothesis would predict. However, with respect to the behavioral results, only in the Stroop task [83] was evidence found in favor of the ‘bilingual advantage in cognitive control’ hypothesis. Finally, in their EEG study, Kousaie and colleagues [53] found no support for the bilingual advantage on a relatedness judgment task in young adults; the analysis of the behavioral scores revealed that monolinguals and bilinguals performed equally well on the task. Only subtle electrophysiological differences in language processing were found. Monolingual adults were found to rely on context to a greater extent than bilingual adults when reading ambiguous words, while bilingual adults showed less selective activation of the contextually appropriate meaning of a homonym than monolingual adults [53].

### 3.4. Experimental Tasks

To see whether a general bilingual advantage in cognitive control exists, the different tasks that are used must be controlled to be able to see whether the same results are received across varying tasks. Therefore, the cognitive control results of the bilingualism studies specified per experimental task are now presented.

#### 3.4.1. Simon Task

As Table 1 shows, Bialystok and colleagues [27] found on the Simon task [80] smaller Simon effect costs for the bilingual group. Furthermore, they found that bilinguals responded more rapidly than monolinguals to conditions that placed greater demands on working memory. In line with this result, Bialystok [29] found in another study with the Simon task that video-game players showed faster responses than other adults under almost all conditions; however, bilingual adults were found to be faster than the video-game players under conditions that required the most controlled attention to resolve conflict. Bialystok and colleagues [31] conducted a third study on both young and older monolingual and bilingual adults and found the greatest levels of control in the older bilingual group, which is also what the ‘bilingual advantage in cognitive control’ hypothesis would predict. In a fourth study with the Simon task, Bialystok and DePape [34] found that both bilingual adults and monolingual musicians performed better than monolingual adults on the Simon task. In line with these results, Schroeder and colleagues [60] also found that bilinguals, musicians, and bilingual musicians showed improved executive control skills compared to monolinguals. Woumans and colleagues [52] also found evidence in favor of the bilingual advantage; bilinguals showed a smaller congruency effect in the Simon task than monolinguals. Cox and colleagues [57] also found that bilinguals outperformed monolinguals. Importantly, the bilingual advantage in conflict processing remained after controlling for the influence of childhood intelligence, the parents’ social class, and the child’s social class. In an MEG study with the Simon task, Bialystok and colleagues [28] found evidence for the hypothesis that the management of two language systems leads to systematic changes in frontal executive functions.

However, not all studies using the Simon task showed a bilingual advantage. Yudes and colleagues [41], for instance, found that interpreters and bilinguals did not outperform monolinguals on the Simon task. Van der Linden and colleagues [68] found similar results; interpreters and L2 teachers did not outperform monolinguals. Paap and Greenberg [21] also found that bilinguals did not outperform monolinguals in either inhibitory control or monitoring; similar results were found in studies by Kousaie and colleagues [48] and by Desjardins and Fernandez [69]. Kirk and colleagues [49] decided to include not only bilinguals, but also bidialectals, in their study; still they found no differences in overall reaction times or in the Simon effect between groups of older bilingual, bidialectal, and monolingual adults.

Other studies with the Simon task found mixed results. Coderre and van Heuven [47] found mixed results, showing the importance of controlling for script similarity of the languages under investigation in studies on the bilingual advantage. Goral and colleagues [55] conducted a study on middle-aged to older adults and found mixed results. On the one hand, dominant bilinguals showed no greater Simon effect with increasing age, which is what the bilingual advantage hypothesis would predict. On the other hand, balanced bilinguals did show a greater Simon effect with increasing age. Struys and colleagues [7] also found mixed results. On the one hand, a higher global accuracy score was found for simultaneous bilinguals compared to early bilinguals, which supports the bilingual advantage. On the other hand, no differences in mean reaction time were found between the two bilingual groups, although that should have been expected when different L2 acquisition between the two groups is considered. In another study by Struys and colleagues [66], again mixed results were found. The two groups of younger and older bilingual children and the two groups of younger and older monolingual children showed no differences in task performance; however, a significant speed–accuracy trade-off across tasks and age groups was found for the bilinguals, but not for the monolinguals. Blumenfeld and Marian [14] found that bilinguals performed worse on the Simon task than on the Stroop task, which was not the case for monolinguals. In an fMRI study by Ansaldo and colleagues [50], no differences in behavioral scores were found between monolinguals and bilinguals in cognitive control performance on the Simon task. However, interestingly, in contrast to elderly monolinguals, elderly bilinguals were found to be able to deal with interference control without recruiting a circuit that would be particularly vulnerable to aging. Kousaie and Phillips [63] also found a discrepancy between the behavioral and the neuroimaging results. On the one hand, no behavioral differences between bilinguals and monolinguals were found, but on the other hand, electrophysiological differences on the Simon task were visible in the data.

Finally, in several studies, methodological factors seem to explain away the possible bilingual advantage scores on the Simon task. For instance, Morton and Harper [30] found no evidence at all for a bilingual advantage when they controlled for socioeconomic status and ethnicity in their study. Naeem and colleagues [67] found faster response times for bilinguals as compared to monolinguals on the Simon task, but that effect vanished when controlled for socioeconomic status.

#### 3.4.2. Attention Network Test

First, Costa and colleagues [33] found that bilinguals were faster on the attention network test [81] than monolinguals. Moreover, they found that bilingual adults were more efficient in alerting and executive control. Bilinguals were found to be better in dealing with conflicting information and to show a reduced switching cost compared to monolinguals. Desideri and Bonifacci [64] showed overall faster reaction times and better conflict performances for bilinguals than for monolinguals. Tao and colleagues [40] showed that both early and late bilinguals performed better on the attention network test than monolinguals, while the best performance was found for early bilinguals. Woumans and colleagues [52] found that bilinguals were faster on the attention network test than monolinguals. Moreover, the error congruency effect was significantly smaller for balanced bilinguals and interpreters in comparison with unbalanced bilinguals and monolinguals. By contrast, Bialystok and colleagues [36] found no differences in scores on the attention network test between bilinguals and monolinguals.

#### 3.4.3. Flanker Task

Emmorey and colleagues [32] had bilingual and monolingual adults perform several Flanker tasks [82]. In their study, both unimodal and bimodal bilingual participants were included. They found no group differences in accuracy; however, unimodal bilinguals were found to be faster than both bimodal bilinguals and monolinguals. Costa and colleagues [35] found that bilingual adults were faster than monolingual adults under a high-monitoring condition, but not under a low-monitoring condition. Engel de Abreu and colleagues [42] found that bilingual children performed better than monolingual children on the Flanker task; this was also reported by Poarch and Bialystok [54]. Moreover, the degree of bilingual experience was not found to play an important role in this bilingual advantage [54]. Xie [65] conducted a study on high-proficiency, middle-proficiency, and low-proficiency bilingual adults and found a better ability on conflict monitoring for the more proficient bilinguals than for the less proficient bilinguals. Struys and colleagues [66] found mixed results in their study. No differences were found between the two groups of younger and older bilingual children compared to the two groups of younger and older monolingual children. However, evidence was found for a significant speed–accuracy trade-off across tasks and age groups for the bilinguals only. Kousaie and Phillips [63] also found mixed results: No behavioral differences between bilinguals and monolinguals were found; however, electrophysiological differences on the Flanker task were visible in the data. In contrast to the previously reported mixed results, Paap and Greenberg [21] found no group differences in their study; bilingual adults and monolingual adults showed similar results on the Flanker task. Moreover, recently, Van der Linden and colleagues [68] found that highly proficient interpreters and L2 teachers did not outperform monolinguals on the Flanker task.

#### 3.4.4. Stroop Task

Bialystok and colleagues [31] used the Stroop task [83] and found that bilingual adults outperformed monolingual adults and that this bilingual advantage was the greatest in the group of older adults. In another study, Bialystok and DePape [34] used the Stroop task again, but this time, they included a group of monolingual musicians in addition to monolingual and bilingual adults. The results of that study showed that the musicians outperformed the monolingual and the bilingual adults on the Stroop task, showing enhanced control in a more specialized auditory task. Blumenfeld and Marian [14] also used a Stroop task and found that bilinguals performed better on the Stroop task than they did on the Simon task [80], which was not the case for monolinguals. Kousaie and colleagues [48] and Kousaie and Phillips [63] also found that bilingual adults showed better scores on the Stroop task than monolingual adults; moreover, the electrophysiological results were found to be different between the bilingual and the monolingual groups [63]. Surprisingly, in contrast to the previous five studies [14,31,34,48,63] in which evidence in favor of the bilingual advantage was found, Duñabeitia and colleagues [46] used a verbal, as well as a nonverbal, Stroop task and failed to find any evidence for the existence of a bilingual advantage. Finally, in their study using the number Stroop task and the N-back task, Dong and Liu [59] discovered that processing demand was a modulating factor for the presence or the absence of bilingual advantages.

#### 3.4.5. Switching Task

Marzecová and colleagues [43] found that on the switching task [36], bilinguals were less affected by the duration of the preceding preparatory interval than monolinguals were. Moreover, bilinguals outperformed monolinguals on the category switch task; reduced switch costs and greater accuracy scores were found. However, Paap and Greenberg [21] found different results; bilingual individuals and monolingual individuals performed similarly on the switching task. Garbin and colleagues [37] conducted an fMRI study in which monolingual and bilingual young adults had to perform a nonlinguistic switching task. They found a reduced switching cost in bilinguals. Moreover, they found that bilinguals activated the left inferior frontal cortex and the left striatum when conducting the nonlinguistic switching task, areas that are known to be involved in language control. Taken together, their results are evidence in favor of a bilingual advantage in cognitive control. In the longitudinal study conducted by Macnamara and Conway [45], a switching task was performed. Their results showed that advanced bilinguals (e.g., interpreter students) outperformed themselves at the second testing after two years. Blanco-Elorrieta and Pylkkänen conducted an MEG study [56] on highly proficient bilinguals, in which they had to perform several switching tasks. Their neuroimaging results showed a clear dissociation of language control mechanisms in production versus comprehension. Only partial support was found for the bilingual advantage hypothesis. Moreover, in another MEG study [62], Blanco-Elorrieta and Pylkkänen showed that switching under external constraints heavily activated prefrontal control regions, but that was not the case for natural, voluntary switching.

#### 3.4.6. Other Experimental Tasks

During the last 15 years, many different experimental cognitive control tasks have been used, in addition to or instead of the previously frequently used cognitive control tasks, in order to investigate the existence of a bilingual advantage. Bialystok and colleagues [36], for instance, used the Luria’s tapping task [90], opposite worlds task [91], and reverse categorization task [92] and found evidence in favor of the bilingual advantage because bilinguals outperformed monolinguals on all three executive functioning tasks. Hsu [44] used a language production task and analyzed the errors and self-repairs of the participants. In the first experiment, a clear advantage in inhibitory control was found for both bilingual and trilingual participants than for monolingual participants. However, in the second experiment, an advantage in attentional control on the production task was only found for the trilinguals. Luo and colleagues [38] used verbal fluency tasks [84] and found more enhanced executive control for bilinguals than for monolinguals on the letter fluency task, but no differences between bilinguals and monolinguals were found on the category fluency task. Teubner-Rhodes and colleagues [58] used an N-back task and found more cognitive flexibility skills; they suggested that this might be the underlying basis for the bilingual advantage. Hsu [61] used a reading task and found that two bilingualism effects dynamically interplayed (depending on the task contexts and the relative degrees of using the first and the second languages) and as a result were affecting the bilingual advantage. In their study, Desideri and Bonifacci [64] used a picture–word identification task, showing evidence for the role of the nonverbal monitoring component in verbal anticipation. On the Wisconsin card sorting test, mixed results have been found so far. On the one hand, Yudes and colleagues [41] found that interpreters outperformed unbalanced-late bilinguals and monolinguals, which is what one would expect based on the bilingual advantage hypothesis. On the other hand, Xie [65] found no differences in scores between the high-proficiency, middle-proficiency, and low-proficiency bilingual groups; similar results were found by Kousaie and colleagues [48], who also found no group differences between bilinguals and monolinguals. Van der Linden and colleagues [68] found no evidence in favor of a bilingual advantage on the N-back task and the Hebb repetition paradigm. They reported only possible advantages in short-term memory. Goral and colleagues [55] found no evidence for a bilingual advantage in alternating attention on the trail making test [94], Kousaie and colleagues [48] found no evidence for a bilingual advantage on the sustained attention to response task [93], and Bialystok and colleagues [31] found no evidence for a bilingual advantage on the sustained attention to response task. On the one hand, Soveri and colleagues [39] found on the dichotic listening task [95] that early simultaneous bilinguals were better than monolinguals in directing attention, as well as in inhibiting task-irrelevant stimuli, supporting the bilingual advantage hypothesis; however, at the same time, Desjardins and Fernandez [69] found no support for the bilingual advantage hypothesis in their dichotic listening data. Surprisingly, Naeem and colleagues [67] even found disadvantages to being bilingual. On the Tower of London task, a monolingual advantage was found, showing higher executive planning abilities in monolinguals than in bilinguals.

In addition to collecting behavioral scores, several studies have collected neuroimaging data, as well. In the Hervais-Adelman and colleagues’ [51] study, multilingual participants performed simultaneous interpretation and repetition tasks in the MR scanner. Brain structures that had previously been found to be active in nonlinguistic executive control tasks were found to be involved, thereby indirectly supporting the bilingual advantage hypothesis. Kousaie and colleagues [53] used a relatedness judgment task and found no evidence of a bilingual advantage. The behavioral scores of bilinguals and monolinguals showed no differences. Only the electrophysiological recordings showed subtle differences in language processing; however, this result neither favored nor disfavored the existence of a bilingual advantage but only showed that monolinguals and bilinguals processed the linguistic information differently.

## 4. Discussion

A systematic review was conducted on bilingualism and cognitive control. First, the study focused on whether the bilingual advantage in cognitive control [43] existed or not. Bilinguals were expected to perform better than monolinguals on cognitive control tasks. Secondly, with respect to the bilingual advantage in cognitive control hypothesis [43], this study was interested in possible modulating factors of this effect. Individual factors, such as socioeconomic status [24], cognitive capacity [25], culture [24], participants’ education level, immigration status [96,97], cultural traits [98], the tremendous variation in linguistic experiences, and interactional contexts, or the specific subcomponents/processes involved in executive functioning [21,46,99,100,101] (see Paradowski [102] for a detailed overview), as well as methodological factors [103], were hypothesized to affect the bilingual advantage.

The first question was whether or not a bilingual advantage in cognitive control existed across studies. In line with our expectation, the results of the present review showed that the majority, 54.3%, of the original studies, indeed found a bilingual advantage in cognitive control; however, at the same time, a substantial number of studies, 28.3%, found mixed results, while 17.4% even found evidence against its existence. In general, the evidence in favor of the existence of a bilingual advantage was stronger in the earlier studies conducted in the period between 2004 and 2012, whereas more mixed findings and studies showing evidence against the existence of a bilingual advantage were found in more recent years, in the period from 2013 until October 2018 (see Figure 3). One explanation for this finding might lie in the improved methodology (e.g., the use of less selective and larger samples, the use of more and different experimental tasks) of the more recently conducted studies [103]. Another explanation might be that open science [104] and publishing null-results [105] have become more popular in recent years, making publishing such data easier. Perhaps the bilingual advantage in cognitive control has been overestimated in the literature in the past [106], but at the same time, this does not mean that the ‘bilingual advantage in cognitive control’ hypothesis is entirely wrong or that a bilingual advantage in cognitive control does not exist [106]. Note that also in the period between 2013 and October 2018, 13 studies found support in favor of its existence versus 10 studies reporting mixed results and 7 studies showing evidence against its existence.

Furthermore, the results obtained from studies investigating adults (56.4%) were found to be more convincingly in favor of the existence of a bilingual advantage in cognitive control than the results obtained from children (42.8%) were. This is an interesting finding. One interpretation could be that the bilingual advantage may not become evident until adulthood. The reason for this difference between bilingual children and bilingual adults might lie in the fact that brain development in children is not yet completed. Especially the ability to perform cognitive control requires the recruitment of prefrontal brain regions [107]. Those regions, however, are not fully developed until early adulthood [107]. Thus, the bilingual advantage in cognitive control may not be as clear and consistent in children due to the fact that their brains are still developing. We should mention, however, that the number of bilingual studies on children in which the bilingual advantage was tested was found to be small, so more future studies on children are definitely needed before any firm conclusions regarding the existence of a bilingual advantage at a young age can be drawn.

Different tasks have been used to test the bilingual advantage in cognitive control. Among them, the Simon task [80], the attention network test [81], Flanker tasks [82], the Stroop task [83], and switching tasks [37] have been most frequently used to test the bilingual advantage in cognitive control, and the results differ across the experimental tasks. The Stroop task results revealed that almost all studies show a bilingual advantage [14,31,34,48,63]. The only exception was a study conducted by Duñabeitia and colleagues [46], but they used both a verbal and a nonverbal Stroop task. On the Flanker task, the majority of studies showed results in favor of a bilingual advantage [32,35,42,54,65] that was visible in better accuracy scores [42,54] and in higher processing speed [32,35], but at the same time, some studies showed more mixed results [63,66], and in two studies, no evidence for a bilingual advantage was found [21,68]. The attention network test results showed a similar picture; the majority of studies showed supporting results [33,40,52,64], with both faster processing speed [33,52,64] and better performance scores being found [40,52,64]. Only one study found no support at all [36]. In contrast to the Stroop task, the Flanker task, and the attention network test results, the results of the Simon task were less clear. Although many studies showed supporting results [27,28,29,31,34,52,57,60], at the same time, almost the same number of studies found mixed results [7,14,47,50,55,63,66]; moreover, a substantial number of studies found evidence against the existence of a bilingual advantage [21,41,48,49,64,68]. The reason for these conflicting results might lie in the fact that the Simon task [80] is too easy to perform and because of the ceiling effect [108], the bilingual advantage often does not appear. On switching tasks, the results were also mixed. Some behavioral results on switching tasks showed a bilingual advantage [43] but not all [21]. In addition, a longitudinal study found that bilinguals perform better over time [45]. In neuroimaging studies in which switching tasks were used, only partial support was found for a bilingual advantage [56]. Finally, the remaining categories of experimental cognitive control tasks, in general, showed mixed results, as well. Some studies showed evidence in favor of a bilingual advantage [36,41,58], while other studies were less clear-cut [38,42]; several studies showed evidence against the existence of a bilingual advantage [48,65,68], and one study even found disadvantages in being bilingual [67]. In sum, more convincing results in favor of the bilingual advantage in cognitive control were found on the Stroop task, the Flanker task, and the attention network test, whereas more heterogeneous and less convincing results regarding its existence were found on the Simon task, switching tasks, and the remaining categories of experimental cognitive control tasks. An explanation for this result might be that both bilingual and monolingual individuals, who are in most cases undergraduate students and young adults, already have maximum scores on the easier cognitive control tasks (e.g., the Simon task [80]) in contrast to the more difficult cognitive control tasks (e.g., the Stroop task [83]). One cannot find any significant differences between bilinguals and monolinguals when both groups have already performed at or near the possible upper limit (ceiling effect) [108]. This might also explain why results in support of a bilingual advantage are often found in older adults [109,110] or in more vulnerable patient groups, such as patients suffering from dementia [111,112] (however, note that some studies reported mixed effects of bilingualism on dementia [77,102]), because here, monolingual control participants do not perform at the maximum, and as a result, the bilingual advantage appears. However, it may also be that lower scores on widely used non-normalized psychometric tests of cognitive ability in older adults do not necessarily reflect decline in cognitive information-processing capacities but higher processing demands (memory search and greater sensitivity to fine-grained differences) due to richer experience and knowledge in older adults [113].

**Figure 4:**
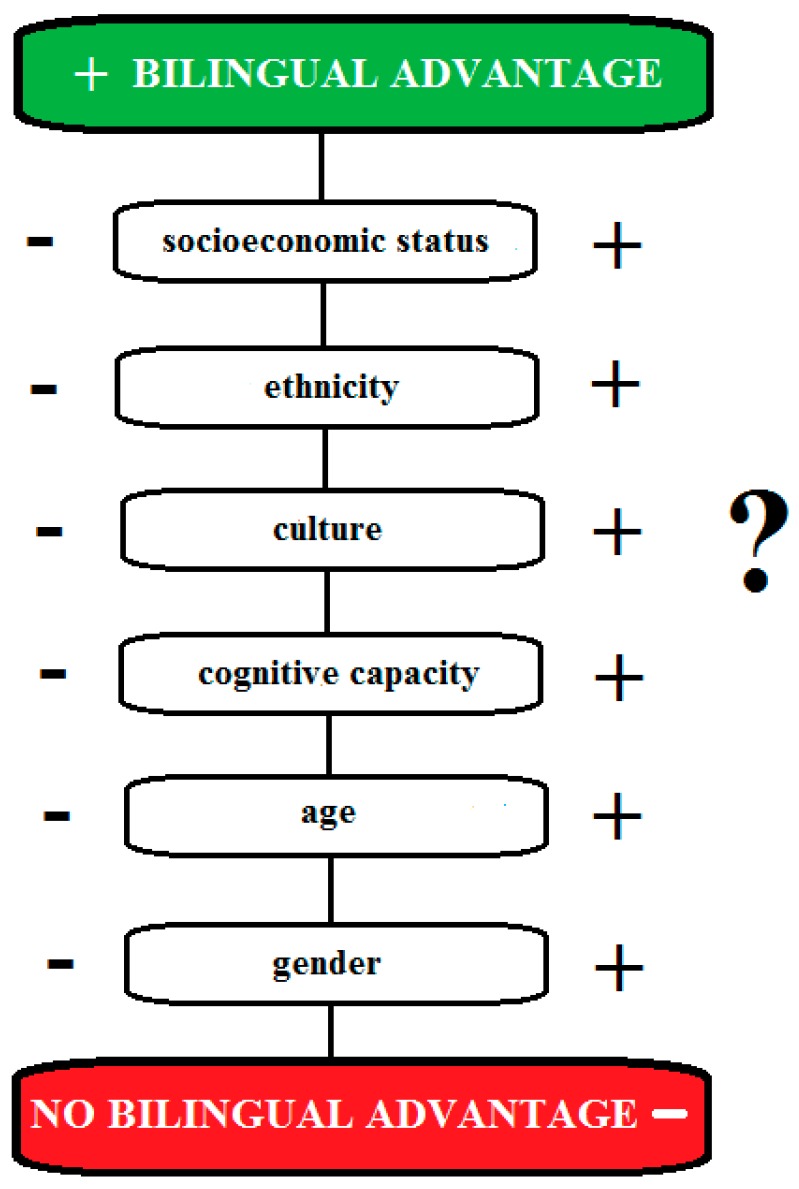
The working model of the bilingual advantage and its modulating factors. The question mark refers to the fact that to date, the strengths of those separate modulating effects remain unclear.

Regarding the second question about the modulating factors of the bilingual advantage in cognitive control, in the literature [45], the interplay between the bilingual management demand and the level of experience the individual has with managing those demands seem to affect the bilingual advantage (Figure 4). Moreover, socioeconomic status [30], ethnicity [30], cultural factors [30,79], processing demand [58], script similarity of the investigated languages [47], and language environment [48] were found to be important modulating factors of the bilingual advantage in cognitive control. In future research, the use of ex-Gaussian distribution analysis [114] in original studies and meta-analyses seems to be a promising approach to investigating better the factors modulating the bilingual advantage in cognitive control. The ex-Gaussian distribution analysis provides a more fine-grained understanding of the different bilingual effects [114]. A detailed discussion of the methodological factors affecting the bilingual advantage is provided below.

### 4.1. General Limitations of Studies Conducted So Far

The current study draws attention to several important limitations of previous bilingual studies that are important to take into account if progress in the research on the bilingual advantage in cognitive control is to be made. For instance, in the research on the bilingual advantage in cognitive control so far, socioeconomic status [30], ethnicity [30], cultural factors [30,79], script similarity of the investigated languages [47], and L2 experience and history [115] seem to be important factors that need to be controlled. For instance, children with less intellectual stimulation during infancy might benefit more in cognitive control from language-switching practice than bilingual children with more intellectual stimulation. Moreover, further research is needed to address whether a high educational level and, as a result, an extended range of cognitive stimulations evens out the bilingual advantage in cognitive control [50]? However, so far, the majority of studies (particularly the older ones) fail to control these factors [30,75]. Moreover, especially for the bilingual advantage studies on older adults, in which experimental tasks with a hearing component, such as the forward and the backward digit span tasks, are involved [48], “age-appropriate hearing” [116] should be controlled for across the subjects in order to be sure that the bilingual advantage results in older adults are not affected by differences in hearing between the bilingual and the monolingual groups of older adults. Some researchers claim that the bilingualism advantage disappears when these modulating factors are controlled [67,75], a claim that has been confirmed in several studies [30,67]. This might be an explanation for the more heterogeneous findings found in recent years (see also Figure 3). However, other researchers [42] have shown a bilingual advantage even after controlling for these factors. For instance, Cox and colleagues [57] found that bilinguals outperformed monolinguals on the Simon task [80] and that the bilingual advantage in conflict processing remained after controlling for the influence of childhood intelligence, the parents’ social class, and the child’s social class. Although this issue is a current topic of debate, from a methodological point of view, clearly these factors must be controlled if any firm conclusions about the existence of a bilingual advantage in cognitive control are to be drawn. Alternatively, one could try to disentangle socioeconomic status issues not by controlling for it but by using it as an independent factor in a, for instance, 2 × 2 (monolingual versus bilingual × low socioeconomic status versus high socioeconomic status) design. Moreover, one must keep in mind that the use of natural group designs [117], which is common in bilingualism research, is a weakness in itself [118,119]. Even when the best control mechanisms possible are applied, the results will never be as reliable as those obtained from laboratory studies. Nevertheless, in general, a need exists for a clear testable working model of the bilingual advantage in order to both move away from the unstructured and chaotic phase that this research field is in at the moment [120] and come to a more scientific approach and structured debate.

Moreover, there might be a publication bias in favor of the bilingual advantage in cognitive control in the literature [73,77], although this is still a matter of debate and no consensus on this issue has been reached [75]. Even though its possible existence would not be unique to this specific field of science (for a detailed discussion, see also the “file drawer problem” in social sciences [121]), it would still be highly problematic. De Bruin and colleagues [73] investigated this publication bias further and found that studies with results fully supporting the bilingual-advantage theory had the highest chance of getting published, followed by studies with mixed results. Studies finding no support for the bilingual advantage, however, were the least likely to be published. This finding cannot be explained by valid scientific reasons, such as differences in sample size, tests used, statistical power, etc. A need exists in science for good-quality journals willing to publish non-effects [122]. This could definitely be beneficial for bilingualism research on cognitive control, could lead to a better overview of the evidence for and against the existence of a bilingual advantage, and as a result, could lead to better and new insights.

Another problem leading to those varying findings between different studies is the fact that they most often do not use standardized test paradigms but instead use all kinds of adaptations of the Simon task [80], the attention network test [81], the Flanker task [82], etc. This is problematic because it makes comparing the bilingual advantage results across different research groups and languages difficult. Due to missing norms, results that have been obtained with nonstandardized tests are hard to interpret correctly. Note that standardized tests are actually designed to compare and rank test takers in relation to one another [123]. In addition to the use of standardized tests, implementing nonlinguistic interference tasks in future research is important in order to test reliably the existence of and the mechanisms behind the bilingual inhibitory control advantage [71]. Unfortunately, a large number of studies failed to do this. Further, small differences in the scoring of the tests between research groups can make significant differences in the outcomes. Zhou and Krott [76], for instance, found that studies that included longer responses in their analysis of the cognitive control tasks were more likely to report a bilingualism effect. Therefore, in future research, this methodological issue should be managed in a better way; in addition, guidelines across research groups should be agreed upon because seemingly insignificant details, such as the data trimming procedure, can have a potential impact on whether the bilingual advantage in cognitive control effect is observed or not [76].

In general, a more integrated approach to cognitive and neuroscience research on the bilingual advantage in cognitive control, instead of working in separate research fields, would seem beneficial for making progress [72]. For instance, previous neuroscience research showed that genetic factors are involved in the working mechanisms of dopamine in the neural structures that underlie the process of cognitive control [74] and revealed new insights about the direction of causality between bilingualism and cognitive control [124]. Recently, a variation in the *DRD2* gene was suggested as having an effect on bilingual verbal and nonverbal cognitive control performance [125]. Moreover, neuroimaging studies on the relation between bilingualism and cognitive control revealed that language control was a subdomain of general executive control in production [56] and that switching under external constraints heavily recruited prefrontal control regions, but that was not the case for natural, voluntarily switching [62]. In addition, the use of neuroimaging methods in research on the relation between bilingualism and cognitive control, in addition to collecting behavioral scores, can provide a more complete picture [126]. Sometimes, no differences are visible in behavioral scores, but the functional and structural neuroimaging results tell a different story [125]. For instance, Kousaie and Phillips [63] found differences in electrophysiological results between bilinguals and monolinguals on all three cognitive control tasks in their EEG study, whereas the behavioral results showed only differences on the Stroop task [83] but not on the Simon [80] and Flanker [82] tasks. A similar discrepancy between behavioral and neuroimaging results was found by Ansaldo and colleagues [50] in their fMRI study. On the one hand, the neuroimaging results supported the bilingual advantage hypothesis, but on the other hand, the behavioral results showed no support for any bilingual advantages in cognitive control. Neuroimaging research can reveal whether bilinguals and monolinguals use different neural pathways (e.g., more efficient, less efficient) during the performance of cognitive control tasks, something that cannot become visible in behavioral studies. Therefore, a more integrated approach might help to build a more complete brain-behavioral model of the bilingual advantage, despite the fact that neuroimaging research (particular fMRI and structural MRI) is expensive and has its own specific methodological difficulties [127]. For instance, differences in the neural activation patterns need not necessarily translate into an advantage. In other words, even if bilingualism does reorganize the brain, such reorganization—or differential neural activation—need not lead to behavioral benefits, and it is not necessarily obvious whether greater effect magnitudes cause/reflect increase or decrease in performance [75].

In addition, foreign language learning is a complex dynamic process [128]. Therefore, bilingual studies with a (short or long-term) longitudinal design [78], taking individual differences more into account [78], are needed in order to tap the dynamics of L2 learning. Only a few longitudinal studies on L2 learning and cognitive control have been conducted so far. Macnamara and Conway [45], for instance, conducted a two-year longitudinal study, showing that the bilingual participants had improved on cognitive abilities associated with managing bilingual demands; however, unfortunately, they failed to include a monolingual control group that received cognitive training via other methods (e.g., musical training, crosswords) in their study. Moreover, in line with the previous point, based on the present studies, how much L2 learning skill one needs to acquire before a bilingual advantage in cognitive control can develop remains unclear. Here, it is important to mention that the nature of the cognitive advantage is gradual, not categorical. Would a minimum amount of active L2 practice [129] already lead to some bilingual advantage in cognitive control or does one need to be a frequent active L2 user? How are the amount of L2 proficiency, active L2 practice, and the degree of the cognitive control advantage exactly related? A determination of the minimum required amount of active L2 practice and minimum required number of L2 skills in order to find some bilingual advantage in cognitive control seems to be beneficial in future research, particularly research using longitudinal designs with different measurements because of the dynamic nature of L2 learning and cognitive control skills.

Another limitation is that, in general, most studies on the bilingual advantage in cognitive control used small sample sizes (e.g., [37,45]) to prove its existence, whereas much larger sample sizes (>138 participants) [130] should have been used in order to achieve desirable levels of statistical power [130,131]. However, at the same time, studies with small sample sizes (e.g., [30,53,69]) were used to prove the opposite, namely, that the bilingual advantage in cognitive control does not exist. Bialystok correctly pointed to this weakness by stating that it is claiming evidence from non-evidence [132]. So far, several studies with large sample sizes have been conducted (e.g., [21,44,46]), but they failed to find a bilingual advantage in cognitive control [21,46]. However, we must point out that those studies used different cognitive control tasks. Therefore, if the bilingual advantage in cognitive control is to be reliably tested and its modulating factors are to be identified, a need exists for big data studies in which similar cognitive control tasks are used (i.e., a whole battery with standardized tests assessing not only the cognitive control domain, but also verbal and nonverbal intelligence, etc.) and the characteristics of the bilinguals and other important factors (e.g., socioeconomic status, ethnicity, cultural differences, age) are measured and controlled.

Given the fact that a majority of studies showed some kind of bilingual advantage in cognitive control (and some disadvantages in lexical access), it seems strange that the usefulness of these cognitive control advantages in classroom settings and for education in general have not been sufficiently investigated [70]. To date, the link between laboratory settings and education has often been missing. How can, in practice, L2 learners and teachers make use of these advantages and, at the same time, take better into account the disadvantages of being bilingual? A recent study by Surmont and colleagues [133], for instance, found that teaching content courses through more than one educational language increased meta-linguistic awareness. The fact that the pupils improved in mathematics more than those who had only been taught in their native language showed that this improved insight extends beyond the linguistic domain. Therefore, future bilingualism research should focus more directly on the educational contexts, as well, in order to deal better with the advantages and disadvantages of being bilingual for education [70].

Finally, surprisingly, the effect of gender is often unaccounted for. Although some studies on the bilingual advantage controlled for gender [64], surprisingly, no bilingualism studies further investigated the effect of gender on cognitive control. This is strange because previous research has shown that gender differences in the neural processes of cognitive control exist [134,135].

### 4.2. Limitations of Our Own Study

Naturally, the present review study has several limitations. The first limitation is that only full data papers and review papers published in internationally peer-review journals were included in this review; no unpublished data or conference materials were included, which differs from what others have done previously [73,77]. This was done to ensure the quality of the included studies. Moreover, some studies that have been presented at several conferences and published in conference proceedings are later published in peer-reviewed journal articles. As a result, these results might be included more often. Because of this methodological decision, analyses of both the effect of publication bias on the data presented in this review and the risk for publication bias were impossible, so the effects of such biases could only be guessed based on other studies.

Secondly, we did not go deeper into the kinds of languages (e.g., language family [136]) spoken by the bilingual participants included in the 46 original studies because many studies simply did not provide such information; thus, this issue is unaccounted for in the presentation of the data. Whether the type of language family plays a role in the appearance of bilingual advantages in cognitive control is a highly interesting issue that needs to be further investigated in future research. So far, recent studies suggest that the advantages reported for ‘true’ multilinguals could be shared by persons speaking two or more dialects of the same language, with children who had developed bidialectal literacy in both the majority and minority written varieties of Norwegian achieving above-national-average scores in standardized tests in reading, arithmetic, and English [137], and bidialectal children speaking both Cypriot Greek and Standard Modern Greek exhibiting an advantage over monolingual peers in holding and manipulating information in working memory [138].

Thirdly, we have decided to present the geographical information about where the original and review studies were conducted in textual instead of tabular form; however, alternatively, one could present this in an additional table. One could argue that the information about the study populations and locations is more substantial than the affiliations of the researchers involved.

## 5. Conclusions

Some evidence was found for a bilingual advantage in cognitive control but not in all studies. Methodological issues and individual differences seem to be important explaining factors for these mixed results. Therefore, better designed, bilingualism studies on cognitive control, particularly big data and longitudinal studies, are needed in order to make progress.

## Figures and Tables

**Table 1:** Overview of the original studies included in the present review. The following information is provided: The authors, the publication year, the citation number, the number of bilingual subjects that participated in the study, the cognitive control tasks that were used, the results of the study, whether the results are in support of, are mixed, or are against the bilingual advantage hypothesis, and the conclusions that were drawn by the authors.

Authors/Publication Year	Number of Bilingual Subjects	Type of Cognitive Control Task	Results	Bilingual Advantage	Conclusions
Bialystok et al., 2004 [27]	20 young adults and 20 older adults	Simon task	Smaller Simon effect costs were found for both the young adult and the older adult bilingual group. Moreover, the bilinguals responded more rapidly to conditions that placed greater demands on working memory than the monolinguals.	YES	The authors conclude that controlled processing is carried out more effectively by bilinguals. Secondly, bilingualism helps to offset age-related losses in certain executive processes.
Bialystok et al., 2005 [28]	20 young adults	Simon task	The MEG results showed that correlations between activated regions and reaction times demonstrated faster reaction times with greater activity in different brain regions in bilinguals compared to monolinguals.	PARTIAL	The management of two language systems led to systematic changes in frontal executive functions.
Bialystok, 2006 [29]	57 young adults	Simon task	Video-game players showed faster responses in almost all conditions; however, bilingual adults were found to be faster than the video-game players in a condition that required the most controlled attention to resolve conflict.	YES	Support was found for the bilingual advantage in cognitive control.
Morton, Harper, 2007 [30]	17 children	Simon task	Bilingual and monolingual children performed identically. Children from higher socioeconomic status families performed better than children from lower socioeconomic status families.	NO	Controlling for socioeconomic status and ethnicity seemed to eliminate the bilingual advantage.
Bialystok et al., 2008 [31]	24 young and 24 older adults	Simon task, Stroop task, Sustained Attention to Response task	Bilinguals performed better than monolinguals on the executive functioning tasks, and this advantage was stronger in the group of older bilinguals. Their working memory performance was the same. The monolinguals outperformed the bilinguals on lexical retrieval tasks.	YES	The executive functioning results are support for the bilingual advantage in cognitive control hypothesis; the bilinguals outperformed the monolinguals.
Emmorey et al., 2008 [32]	30 middle-aged adults	Flanker tasks	No group differences in accuracy were found. However, the unimodal bilinguals were faster than the bimodal bilinguals and the monolinguals.	PARTIAL	The bilingual advantage in cognitive control is the result of the unimodal bilingual’s experience controlling two languages in the same modality.
Costa et al., 2008 [33]	100 young adults	Attention Network Test	Bilinguals were faster on the attention network test than the monolinguals; moreover, they were more efficient in alerting and executive control. Bilinguals were better in dealing with conflicting information and showed a reduced switching cost as compared to the monolinguals.	YES	Bilinguals have more efficient attentional mechanisms than monolinguals. This finding supports the bilingual advantage hypothesis.
Bialystok, DePape, 2009 [34]	24 young adults	Simon task, Stroop task	The bilingual adults and monolingual musicians performed better than the monolingual adults on the Simon task. Moreover, the monolingual musicians outperformed the monolingual and bilingual adults on the Stroop task.	YES	The results on the Simon task are support for the bilingual advantage. In addition, musicians were found to have enhanced control in a more specialized auditory task; this was not the case for the bilingual adults.
Costa et al., 2009 [35]	122 young adults	Flanker task	The bilinguals were faster than the monolinguals in the high-monitoring condition, but not in the low-monitoring condition.	YES	Support was found for the hypothesis that bilingualism may affect the monitoring processes involved in executive control.
Bialystok et al., 2010 [36]	56 children	Attention Network Test, Luria’s tapping task, Opposite Worlds task, reverse categori- zation task	The bilingual children performed better on the Luria’s tapping task, opposite worlds task, and reverse categorization task than the monolingual children. On the attention network test, no differences in scores between the bilingual and the monolingual children were found.	YES	Evidence was found for a bilingual advantage in several aspects of executive functioning in young children. This bilingual advantage is present at an earlier age than was previously reported in the literature.
Garbin et al., 2010 [37]	19 young adults	Nonlinguistic Switching task	A reduced switching cost was found in the bilinguals. The bilinguals activated the left inferior frontal cortex and the left striatum, areas that are known to be involved in language control.	YES	The early training of bilinguals in language switching (back and forth) leads to the activation of brain regions known to be involved in language control when conducting nonlinguistic cognitive tasks.
Luo et al., 2010 [38]	40 young adults	Verbal fluency tasks	The letter fluency results showed enhanced executive control for bilinguals compared to monolinguals. No differences between bilinguals and monolinguals were found in category fluency.	YES	The bilinguals showed enhanced executive control on the letter fluency task, supporting the bilingual advantage hypothesis.
Soveri et al., 2011 [39]	33 adults varying from young to older	Dichotic listening task	Early simultaneous bilinguals outperformed the monolinguals in the forced-attention dichotic listening task; better scores in the forced-right and forced-left attention conditions were found.	YES	Early simultaneous bilinguals are better than monolinguals in directing attention and in inhibiting task-irrelevant stimuli, supporting the bilingual advantage hypothesis.
Tao et al., 2011 [40]	66 young adults	Attention Network Test	Both early and late bilinguals had an advantage in conflict resolution compared to monolinguals; the greatest advantage was found for the early bilinguals.	YES	Specific factors of language experience may affect cognitive control differently.
Yudes et al., 2011 [41]	32 young to middle-aged adults	Simon task, Wisconsin Card Sorting Test	Simultaneous interpreters showed better cognitive flexibility scores than bilinguals and monolinguals; however, no differences in inhibition scores were found.	PARTIAL	Some evidence in favor of the bilingual advantage was found. Interpreters indeed outperformed the monolinguals in cognitive flexibility. However, the inhibition results showed a different picture; the interpreters, bilinguals, and monolinguals showed similar results, which is not what the bilingual advantage hypothesis would predict.
Engel de Abreu et al., 2012 [42]	40 children	Complex and simple WM tasks, selective attention test, Flanker task	The bilinguals were better than the monolinguals in cognitive control.	YES	The bilingual advantage was found after controlling for socioeconomic and cultural factors. The bilingual advantage was found for cognitive control and not in other domains.
Marzecová et al., 2013 [43]	22 young adults	Switching tasks	Bilinguals were found to be less affected by the duration of the preceding preparatory interval compared to monolinguals. Moreover, bilinguals outperformed monolinguals on the category switch task; reduced switch costs and greater accuracy scores were found.	YES	Bilingualism was positively found to influence the mechanisms of cognitive flexibility.
Paap, Greenberg, 2013 [21]	122 young adults	Simon task, Flanker task, Switching task	No evidence was found for consistent cross-task advantages in executive processing for the bilinguals compared to the monolinguals.	NO	No consistent cross-task correlations were found, showing evidence against the existence of a bilingual advantage in executive processing.
Hsu, 2014 [44]	78 young adults	Speech production tasks	The first experiment showed that bilinguals and trilinguals outperformed monolinguals in all aspects of inhibitory control. The second experiment showed only an advantage in attentional control for the trilinguals.	YES	The advantage in inhibitory control was visible in more contexts for the trilinguals than for the bilinguals.
Macnamara, Conway, 2014 [45]	21 young adults	Switching task, Mental flexibility task, WM tasks	The adult bimodal bilinguals were followed and re-tested for two years. During this time, their cognitive abilities associated with managing the bilingual demands improved.	YES	The mechanisms recruited during bilingual management and the amount of experience managing the bilingual demands are underlying factors of the bilingual advantage on cognitive control.
Duñabeitia et al., 2014 [46]	252 children	Stroop task	No differences in inhibitory performance scores were found between the bilingual and the monolingual children.	NO	No evidence was found for a bilingual advantage on simple inhibitory tasks.
Coderre, van Heuven, 2014 [47]	58 young adults	Simon task, Stroop task	The similar-script bilinguals were found to have more effective domain-general executive control than the different-script bilinguals.	PARTIAL	No consistent evidence for a bilingual advantage was found, only global response time effects. Script similarity is an important variable to control.
Blumenfeld, Marian, 2014 [14]	90 young adults	Simon task, Stroop task	The bilinguals performed better on the Stroop task than on the Simon task. The monolinguals did not perform differently on the two cognitive control tasks.	YES	Evidence was found for a bilingual advantage in cognitive control where bilingualism may be especially likely to modulate cognitive control mechanisms resolving the stimulus–stimulus competition between two dimensions of the same stimulus.
Kousaie et al., 2014 [48]	51 young adults and 36 older adults	Simon task, Stroop task, Sustained Attention to Response task, Wisconsin Card Sorting Test	In some executive functioning tasks, the bilinguals outperformed the monolinguals, but these findings were not consistent across executive function tasks. Moreover, no disadvantage was found for bilinguals on language tasks. Finally, evidence was found that language environment might be an important modulating factor.	PARTIAL	Although in some executive functioning tasks, the bilinguals do outperform the monolinguals, these findings are not consistent across tasks. Language environment seems to be an important modulating factor.
Kirk et al., 2014 [49]	32 older adults	Simon task	The bilinguals, bidialectals, and monolinguals showed no differences in overall reaction times or in the Simon effect.	NO	No evidence was found for a bilingual or bidialectal advantage in executive control.
Ansaldo et al., 2015 [50]	10 older adults	Simon task	No differences in behavioral scores between the monolinguals and the bilinguals in cognitive control performance were found. However, interestingly, in contrast to the elderly monolinguals, the elderly bilinguals were found to deal with interference control without recruiting a circuit that is particularly vulnerable to aging.	PARTIAL	On the one hand, the neuroimaging results are support for the bilingual advantage hypothesis; on the other hand, the behavioral results show no support for any bilingual advantages in cognitive control.
Hervais-Adelman et al., 2015 [51]	50 young adults	Simultan- eous inter- pretation and repetition	The caudate nucleus was found to be implicated in the overarching selection and control of the lexicosemantic system in interpretation while the putamen was found to be implicated in ongoing control of language output.	YES	A clear dissociation of specific dorsal striatum structures in multilingual language control was found areas that are known to be involved in nonlinguistic executive control.
Woumans et al., 2015 [52]	93 young adults	Simon task, Attention Network Test	The bilingual participants showed a smaller congruency effect in the Simon task and were overall faster on the attention network test in comparison with the monolinguals.	YES	Support was found for the bilingual advantage; moreover, different patterns of bilingual language use affect the nature and extent of this advantage.
Struys et al., 2015 [7]	34 children	Simon task, verbal fluency task	A higher global accuracy score was found on the Simon task for the simultaneous bilingual children compared to the early bilingual children. No differences in mean reaction time were found between the two bilingual groups.	PARTIAL	No advantage in terms of verbal fluency was found. However, simultaneous bilingual children have an advantage on the Simon task, even over early bilingual children and when L2 is controlled.
Kousaie et al., 2015 [53]	17 young adults	Stroop task, Animacy Judgment task, lexical ambiguity task	No behavioral differences between the bilingual and the monolingual adults were found. However, subtle processing differences were visible in the electrophysiological data.	NO	Monolinguals rely more on context in the processing of homonyms, while bilinguals simultaneously activate both meanings.
Poarch, Bialystok, 2015 [54]	143 bilingual children	Flanker task,	The bilinguals showed better scores than the monolinguals on the conflict trials in the Flanker task. The degree of bilingual experience was not found to play an important role.	YES	Evidence was found for a bilingual advantage in executive functioning. Moreover, the degree of bilingualism experience does not seem to play an important role in this bilingual advantage.
Goral et al., 2015 [55]	106 middle-aged to older adults	Simon task, Trail Making test	Balanced bilingual adults showed a greater Simon effect with increasing age, but this was not the case for the dominant bilingual adults.	PARTIAL	Mixed results were found. On the one hand, the results of the dominant bilinguals support the bilingual advantage hypothesis; on the other hand, the results of the balanced bilinguals showed age-related inhibition decline.
Blanco-Elorrieta, Pylkkänen, 2016 [56]	19 young adults	Switching tasks	The bilingual results show a clear dissociation of language control mechanisms in production versus comprehension.	PARTIAL	Partial support was found for the bilingual advantage; language control is a subdomain of general executive control in production.
Cox et al., 2016 [57]	26 bilingual older adults	Simon task	The bilinguals outperformed the monolinguals on the Simon task. This bilingual advantage in conflict processing remained after controlling for the influence of childhood intelligence, as well as the parents’ and the child’s social class.	YES	Evidence was found for the bilingual advantage in the cognitive control hypothesis. L2 learning was found to be related to better conflict processing. Moreover, neither initial childhood ability nor social class was found to be a modulating factor.
Teubner-Rhodes et al., 2016 [58]	59 young adults	N-back task	Bilinguals performed better than monolinguals on a high-conflict task; however, this was not the case on a no-conflict version of the N-back task and on sentence comprehension.	YES	Evidence was found for the bilingual advantage. This advantage may suggest better cognitive flexibility skills.
Dong, Liu, 2016 [59]	145 young adults	Stroop task, switching task, N-back task	The bilinguals with interpreting experience showed improvements in switching and updating performance, while the bilinguals with translating experience showed only marginally significant improvements in updating.	YES	Processing demand was found to be a modulating factor for the presence or absence of bilingual advantages.
Schroeder et al., 2016 [60]	112 young adults	Simon task	The bilinguals, musicians, and bilingual musicians showed improved executive control skills compared to the monolinguals.	YES	Evidence was found for the existence of a bilingual advantage in executive control as well as for musicians.
Hsu, 2017 [61]	64 young to middle-aged adults	A reading task	The balanced and unbalanced bilinguals were better than the monolinguals on the noncontextual single-character reading task (regardless of their first language background) but not on the contextual multiword task. Finally, the unbalanced bilinguals performed better on the noncontextual task than the other two groups.	YES	The two bilingualism effects dynamically interplayed (depending on the task contexts and the relative degrees of using the first language and L2), and both affected the bilingual advantage.
Blanco-Elorrieta, Pylkkänen, 2017 [62]	19 young adults	Switching tasks	The results of the bilinguals showed that switching under external constraints heavily recruited prefrontal control regions. This result is in sharp contrast with natural, voluntary switching when the prefrontal control regions are less recruited.	PARTIAL	Partial evidence was found for the bilingual advantage. This was only visible when bilinguals needed to control their languages according to external cues and not when switching was fully free.
Kousaie, Phillips, 2017 [63]	22 older adults	Stroop task, Simon task, Flanker task	Bilinguals outperformed the monolinguals on the Stroop task, but no behavioral differences on the Simon and the Flanker task were found. Moreover, electrophysiological differences on all three experimental tasks were found between the bilinguals and the monolinguals.	PARTIAL	Mixed results were found. Group differences in electrophysiological results on all cognitive control tasks between the bilinguals and monolinguals were found. However, only the behavioral results on the Stroop task supported the bilingual advantage in the cognitive control hypothesis.
Desideri, Bonifacci, 2018 [64]	25 young to middle-aged adults	Attention Network Test, Picture-word identifica-tion task	The bilingual adults showed overall faster reaction times and a better conflict performance. Moreover, evidence was found for a role of the nonverbal monitoring component on verbal anticipation.	YES	Bilinguals were found to have more efficient reactive processes than monolinguals. Moreover, support was found for a role of the nonverbal monitoring component on verbal anticipation.
Xie, 2018 [65]	94 young adults	Flanker task, Wisconsin Card Sorting Test	The Flanker results revealed a better ability of conflict monitoring for the more proficient bilinguals. The Wisconsin card sorting test showed no differences between the high-proficiency, middle-proficiency, and low-proficiency bilingual groups.	PARTIAL	The degree of L2 proficiency was found to affect conflict monitoring but had no influence on inhibition or mental set shifting.
Struys et al., 2018 [66]	59 children	Simon task, Flanker task	The bilinguals performed similarly on the two cognitive control tasks compared to the monolinguals. However, only the bilinguals showed a significant speed–accuracy trade-off across tasks and age groups.	PARTIAL	Differences in strategy choices were found to be able to mask variations in performance between bilingual children and monolingual children, leading to inconsistent findings on the bilingual advantage in cognitive control.
Naeem et al., 2018 [67]	45 young adults	Simon task, Tower of London task	Bilinguals were found to have shorter response times on the Simon task, without getting higher error rates. However, socioeconomic status was an important modulator of this effect. Interestingly, a monolingual advantage on the Tower of London task was found, showing higher executive planning abilities.	NO	Evidence was found against a broad bilingual advantage in executive function. Social economic status was found to be an important modulator.
Van der Linden et al., 2018 [68]	25 middle-aged adults	Flanker task, Simon task, N-back task, Hebb repetition paradigm, Digit span task	The highly proficient bilinguals (interpreters and L2 teachers) did not outperform the monolinguals with respect to interference suppression, prepotent response inhibition, attention, updating, and short-term memory.	NO	No evidence was found for general cognitive control advantages in highly proficient bilinguals. Only possible advantages in short-term memory were reported.
Desjardins, Fernandez., 2018 [69]	19 young adults	Dichotic listening task, Simon task	No differences in scores on any of the dichotic listening conditions were found between the bilinguals and the monolinguals. Moreover, no group differences on the visual test of inhibition were found.	NO	No evidence was found for a bilingual advantage in the inhibition of irrelevant visual and auditory information.
